# Intra-articular dislocation of patella with femoral impaction—A case report and review of literature

**DOI:** 10.1016/j.ijscr.2019.05.041

**Published:** 2019-05-29

**Authors:** A. Al Khalifa, K. Syed

**Affiliations:** Toronto Western Hospital, Toronto, ON, Canada

**Keywords:** Case report, Intra-articular, Patella, Dislocation

## Abstract

**Introduction and case presentation:**

Acute traumatic patella dislocations are common injuries seen in emergency departments. We present a rare injury which involves an acute, irreducible intra-articular dislocation of the patella. In this particular case, we describe a horizontal type intra-articular dislocation of the patella in a 66-year-old lady, which was complicated by impaction into the distal femur. She underwent two failed closed reduction attempts, but subsequently reduced spontaneously on the orthopaedic floor as she was waiting for an open reduction in the operating room.

**Discussion:**

The management of acute patellar dislocations can be divided into closed and open reduction methods. In cases where a closed reduction is unsuccessful, an open reduction under general anesthesia may be pertinent to treat this acute condition.

**Conclusion:**

We report a case of an intra-articular patella dislocation which has shown that spontaneous reduction is possible for cases following unsuccessful initial closed reduction, yet it still remains unadvisable to delay open reduction. We highlight, through this case, the importance of maintaining a high index of suspicion for these uncommon injuries.

## Highlights

• Intra-articular patellar dislocations are a rare subtype of traumatic patella dislocation.
• This case report highlights the possibility of these dislocations reducing spontaneously while immobilized in an extension brace.
• It is essential to evaluate for extensor mechanism disruption with such injuries.

## 1 Introduction

Traumatic acute patellar dislocation is a common injury seen in the emergency departments with the most common subtype involving lateral dislocation of the patella. Sports and dance are described as the main activities associated with this type of injury. With regards to the rare occurrence of an intra-articular dislocation of the patella, two types have been described [1]. We present a case of horizontal dislocation of patella with impaction into the distal femur in a 66-year-old patient after trauma.

The following case report has been reported in line with SCARE criteria [2].

## 2 Case history

A 66-year-old female presented to the emergency department after sustaining a mechanical fall earlier that day. She reported that she had missed a step on the sidewalk, which resulted in a twisting movement to her left knee and direct impact on the floor.

**Fig. 1:**
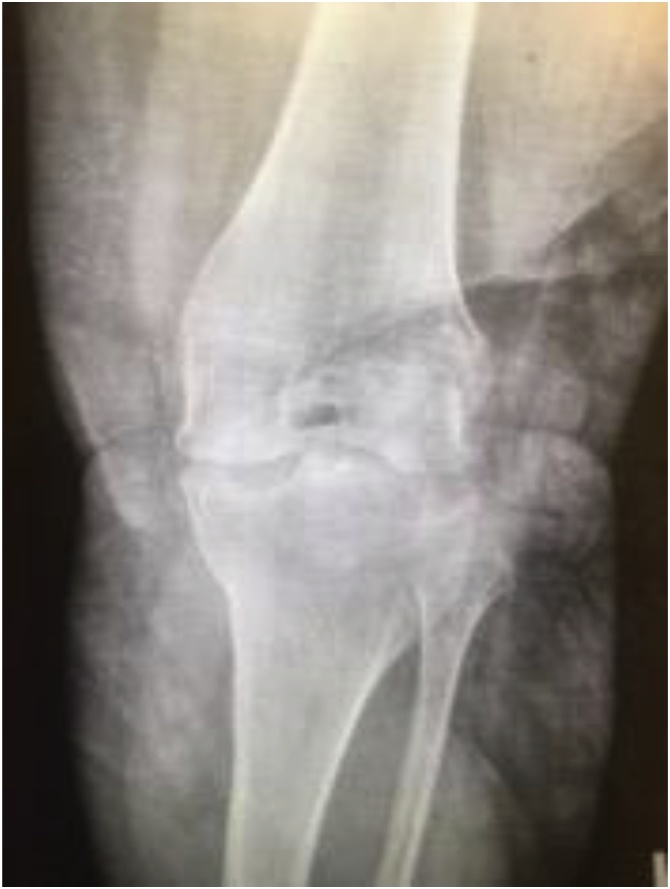
Left Knee AP Radiograph.

**Fig. 2:**
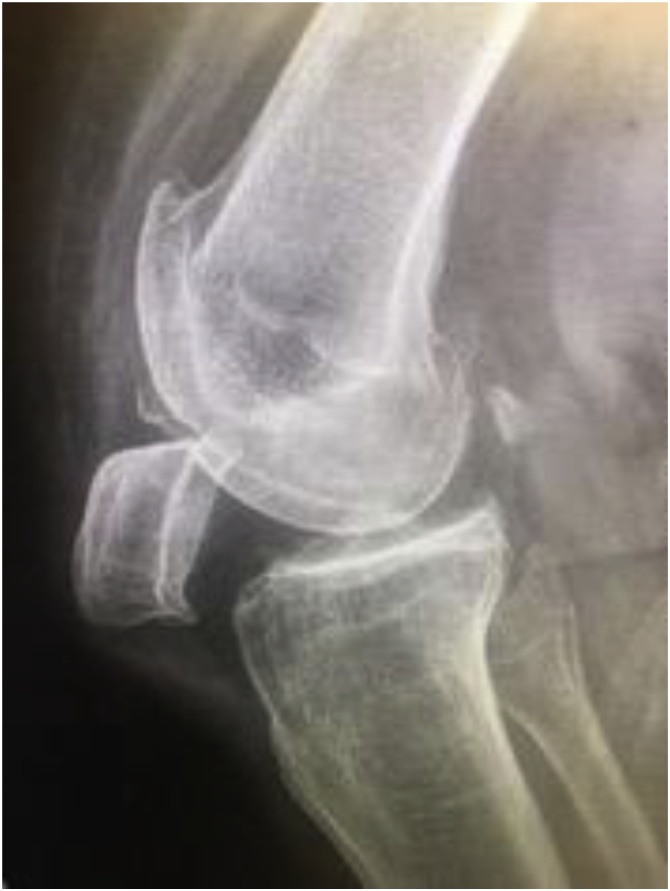
Left Knee Lateral Radiograph.

On examination, her left knee was found to be locked in approximately 60 degrees of flexion and upper pole of the left patella was not palpable. The patellar tendon was intact on palpation, but the quadriceps tendon was not easily palpable. Radiographs of the left knee (Figs. 1 & 2 ) revealed an intra-articular dislocation of the patella with possible impaction into the distal femur.

**Fig. 3:**
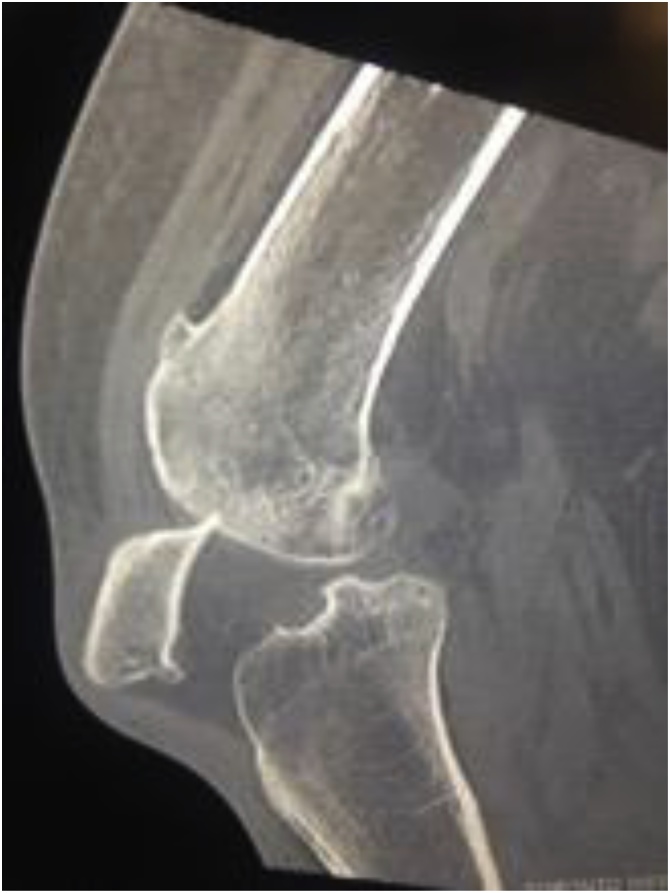
Left Knee CT (Sagittal).

**Fig. 4:**
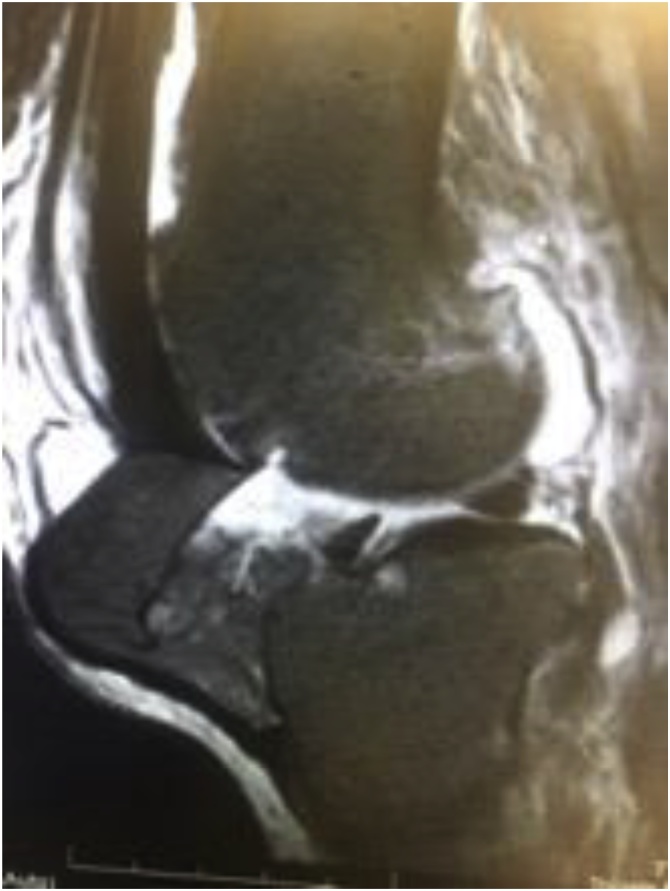
Left Knee MRI (T2).

An unsuccessful closed reduction under procedural sedation was performed in the emergency department. During this maneuver, an attempt was made to reduce the patella with the knee in both extension and flexion but was unsuccessful. A CT of the left knee was obtained following this attempt, which confirmed impaction of the patella into the distal femur (Fig. 3). The patient was then consented for a left patella closed vs open reduction in the operating room. A pre-operative MRI of the left knee confirmed an intact quadriceps tendon (Fig. 4). She was placed in a zimmer (extension) splint for comfort while waiting for the operation.

**Fig. 5:**
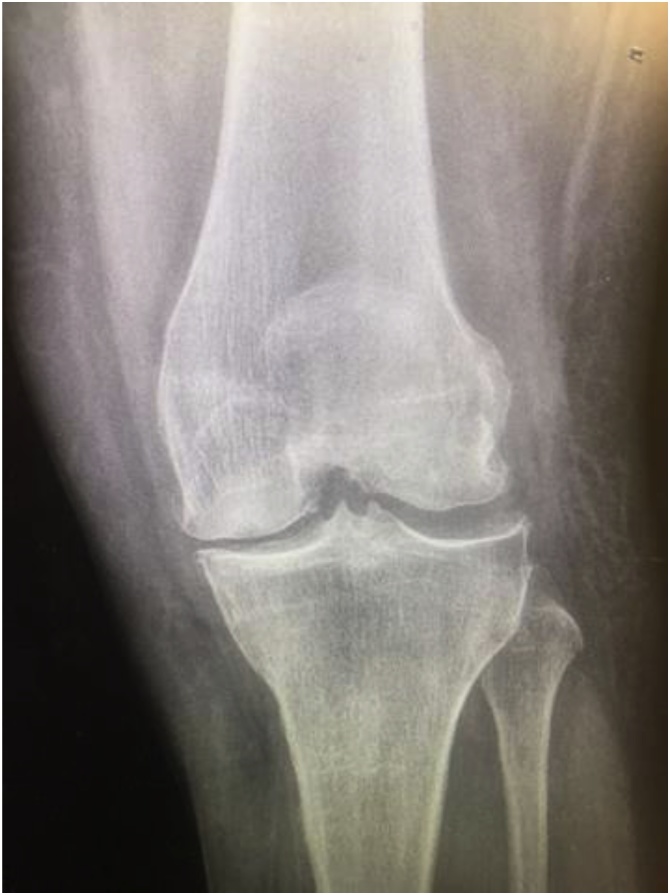
Left Knee AP Radiograph (Post Reduction).

**Fig. 6:**
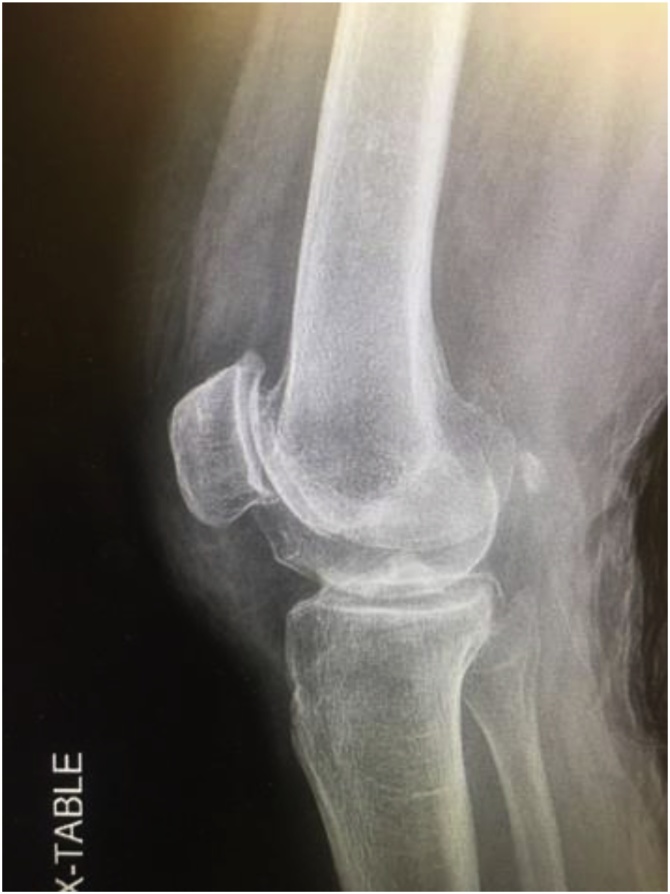
Left Knee Lateral Radiograph (Post Reduction).

The patient was re-examined on the orthopaedic floor on the following day and was found to have spontaneously reduced while she was on the zimmer splint. Post reduction radiographs were obtained to confirm this (Figs. 5 & 6 ). Patient was advised to walk weight-bearing as tolerated in the zimmer (extension) brace for 2 weeks, and has since returned to preinjury mobility.

## 3 Discussion

Incidence of traumatic patella dislocation is very common, with lateral dislocations comprising most of these injuries. The most probable mechanisms described for such injuries involve either a direct blow to the patella or an injury in a flexed, valgus knee position with internal rotation of the femur [3]. Table 1 describes the different classifications of patellar instability with a brief description of each [4].

**Table 1:** Patellar Instability Classification.

Terminology	Description
Patellar Subluxation	Partial movement of patella out of trochlea with presence of some contact between patella and trochlea
Patellar Dislocation	Complete displacement of patella out of trochlea
First-time Patellar Dislocation	First true episode of dislocation wherein the deformity had to be reduced
Recurrent Patellar Dislocation	Subsequent episodes of dislocation wherein the deformity had to be reduced
Passive Patellar Dislocation	Dislocatable patella with an apprehension test
Habitual Patellar Dislocation	Involuntary dislocation and relocation with every cycle of knee flexion and extension
Congenital Patellar Dislocation	Intrauterine patella dislocation with associated characteristic limb deformities
Developmental Patellar Dislocation	Patellar instability not present at birth but develops after walking age
Voluntary Patellar Dislocation	Patellar dislocation and relocation that can be demonstrated by selective muscle contraction without significant knee movement
Syndromic Patellar Dislocation	Patellar dislocation associated with neuromuscular disorder, connective tissue disorder, or syndrome

Intra-articular patella dislocations are considered to be quite uncommon variants of such injuries, and are commonly described to be due to a direct blow onto the patella in a flexed knee position. It involves two subtypes – horizontal and vertical, depending on the patella axis of rotation during the traumatic incident [5]. The quadriceps tendon may rupture completely or partially, especially in a horizontally oriented intra-articular patella dislocation [6].

Two age groups are typically described regarding intraarticular patellar dislocations: adolescents and elderly. In adolescents, it is important to recognize and be aware of the possibility of concomitant sleeve fracture of the superior pole in association with such an injury [7].

The main method of reduction described with regards to the horizontal type intra-articular patellar dislocation involves initial hyperextension of the knee joint followed by passive flexion with upwards pressure over the patella. In our case described above, we were unable to reduce the patella initially due to its impaction into the distal femur. Other factors affecting reduction of intraarticular patellar dislocations include superior osteophytes on upper pole of patella in arthritic knees, inadequate sedation or pain control during reduction maneuver, and strong quadriceps function [8].

Upon reviewing the literature, no case reports were found to have resulted in spontaneous reduction of an intra-articular patellar dislocation. This is most likely due to the fact that most cases were either reduced using the closed method or taken early to the operating room for an open reduction. We hypothesize that our case resulted in spontaneous reduction as a result of quadriceps muscle fatigue, which resulted in decreased traction on the patella with less impaction force into the distal femur and therefore spontaneous reduction.

In cases where a closed reduction is unsuccessful, an open reduction under general anesthesia may be pertinent to treat this acute condition. Gavin McHugh et al. [9]. reported one case which required an open reduction to reduce an intra-articular patellar dislocation following failed closed attempts in the emergency department. They managed to achieve this by performing a midline longitudinal knee incision followed by utilizing a para-quadricipital approach to access the superior pole of patella.

## 4 Conclusion

This case highlights the importance to maintain a high index of suspicion for these uncommon injuries. It should be considered to be an entity amongst the differential diagnosis of a locked knee in the elderly population. Recovery and rehabilitation are more efficient with cases that present without quadriceps or patellar tendon injuries, as these may require further immobilization of the knee joint.

## Conflicts of interest

None.

## Sources of funding

None needed.

## Ethical approval

Exempt from ethical approval at UHN, Toronto, CA.

## Consent

Written informed consent was obtained from the patient for publication of this case report and accompanying images. A copy of the written consent is available for review by the Editor-in-Chief of this journal on request.

## Author’s contribution

Ahmed Al Khalifa – writing paper, data collection, literature review, data analysis, manuscript editing, manuscript finalization

K. Syed – manuscript editing and review.

## Registration of research studies

N/A.

## Guarantor

Al Khalifa.

## Provenance and peer review

Not commissioned, externally peer-reviewed.
